# Laparoscopic placement of a tined lead electrode on the pudendal nerve with urodynamic monitoring of bladder function during electrical stimulation: an acute experimental study in healthy female pigs

**DOI:** 10.1186/2193-1801-3-309

**Published:** 2014-06-24

**Authors:** Elena E Foditsch, Bogdan Hoinoiu, Günter Janetschek, Reinhold P Zimmermann

**Affiliations:** Spinal Cord Injury and Tissue Regeneration Center Salzburg, Strubergasse 21, 5020 Salzburg, Austria; Pius Branzeu Centre for Laparoscopic Surgery and Microsurgery, Victor Babes University of Medicine and Pharmacy Timisoara, P-ta Eftimie Murgu Nr. 2, 300041 Timisoara, Romania; Department of Urology and Andrology, Salzburg General Hospital, Muellner Hauptstraße 48, 5020 Salzburg, Austria

**Keywords:** Laparoscopy, Electrode implantation, Pudendal nerve, Neuromodulation, Histology, Pig

## Abstract

**Purpose:**

The aim of this study was to develop a method for standard laparoscopic access to the pudendal nerve in pigs to implant an electrode for chronic neuromodulation studies.

**Methods:**

Using routine laparoscopic surgical techniques, the pudendal nerve was located in 10 female pigs using standardized anatomical landmarks. A tined lead electrode was placed in parallel to the exposed pudendal nerve, and acute unilateral electrical stimulation was performed consecutively on both pudendal nerves. Bladder pressure and perineal skeletal muscle response was monitored during stimulation.

**Results:**

Standard access to the pudendal nerve was successfully established in the pig model with surgical times of approximately 45 minutes for bilateral electrode placement. Acute unilateral stimulation did not evoke bladder responses but resulted in reliable stimulation-dependent activity of the perineal skeletal muscles. The structural integrity of the pudendal nerves was confirmed in all cases.

**Conclusions:**

These results illustrate the effectiveness of laparoscopy for standardised, safe nerve localisation and electrode implantation at the pudendal nerve in pigs. Laparoscopic implantation represents an alternative approach for performing electrode implantation under optical guidance versus the standard approach of percutaneous, neuro-physiological monitored implantation. In the future, pudendal neuromodulation may be used as a supplement to sacral neuromodulation or as a standalone therapeutic approach, depending on the underlying bladder dysfunction.

## Background

The pudendal nerve is a peripheral nerve composed mainly of afferent sensory fibres from sacral nerve roots S1, S2, and S3. Consequently, the pudendal nerve is a major contributor to afferent regulation of bladder function (Peters 2010). Due to the large number of afferent fibres, the pudendal nerve is an attractive target for neuromodulation therapy for patients affected by bladder dysfunctions such as overactive bladder or urinary incontinence (Le and Kim 2011). Various sites have been used for electrical stimulation of the pudendal nerve. Specifically, early studies that sought to manage incontinence by neuromodulation mainly used perineal, transvaginal, or rectal stimulation of branches of the pudendal nerve. The current method of choice for pudendal neuromodulation is percutaneous implantation of a tined lead electrode at the pudendal nerve using either a posterior or perineal approach (Peters et al. 2005; Spinelli et al. 2005; Martens et al. 2011). Although the percutaneous implantation method is highly feasible and has been tested in clinical trials (Spinelli et al. 2003), an alternative implantation technique is needed for patients in whom a percutaneous approach is problematic or impossible. Here we tested a minimally invasive technique in which an electrode was guided laparoscopically to the pudendal nerve using specific anatomical landmarks.

Laparoscopy is a highly precise and minimally invasive surgical technique that is the gold standard approach for many surgical interventions. Laparoscopic implantation may have advantages, such as lower electrode migration risk and secured placement under optical control with improved stimulus transmission. Although Possover et al. (2010) have demonstrated laparoscopic implantation of electrodes to various nerves in the small pelvis, the current study focuses exclusively on the pudendal nerve as one of the main regulators of bladder function. Having an alternative method for surgical electrode placement near the pudendal nerve would be valuable for functional modulation of the dysfunctional bladder.

## Results and discussion

### Surgery

**Figure 1:**
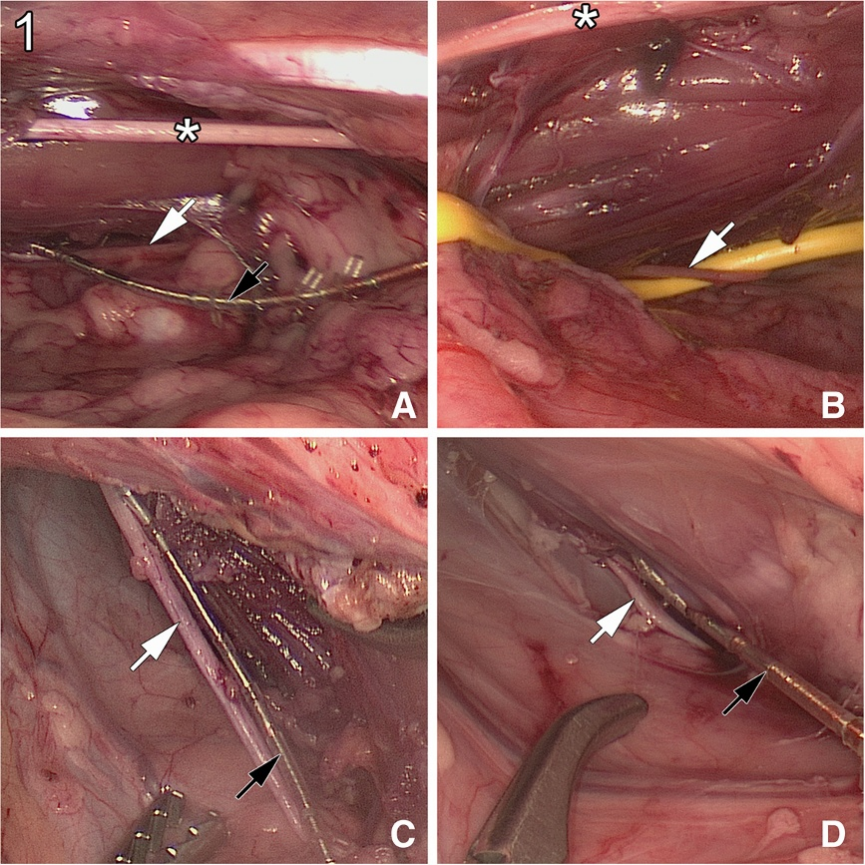
**Laparoscopic access to the porcine pudendal nerve. A**, **B**: Ventral top views of the obturator nerve (*), the pelvic side wall, and, after opening the fascia, the dorsally located pudendal nerve (white arrow). In panel **B**, a vessel rein is used to fix the pudendal nerve. **C**, **D**: Photos of the pudendal nerve with tined lead electrodes (black arrows) placed in parallel to the nerve for acute stimulation.

The surgeries were completed successfully in all 10 pigs. The operating time for bilateral electrode placement was approximately 45 minutes. No intraoperative complications were identified. Standardised localization of the pudendal nerve and placement of the electrode at the nerve site was successful in all animals (Figure 1). The tined lead electrode was unsatisfactory for laparoscopic handling due to its rigidness. Stimulation success was judged by response of the perineal skeletal muscle. During stimulation, contraction of the perianal skeletal muscle was clearly visible.

### Urodynamics

**Figure 2:**
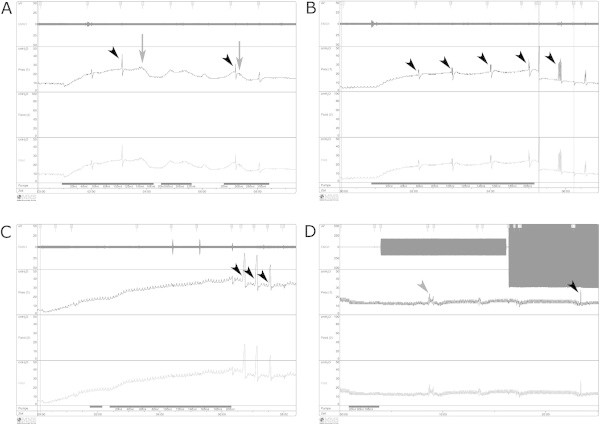
**Urodynamic data from a single animal.** Measurements were performed during light sedation (**A**; control measurement), general anaesthesia **(B)**, after creation of the pneumoperitoneum **(C)**, and during unilateral stimulation **(D)**. Spontaneous voiding (arrows) was seen only in the control measurement **(A)**; there was no spontaneous voiding after deepening of the anaesthesia **(B-C)**. No bladder response was observed during stimulation using either low stimulation parameters or high stimulation parameters **(D)**. Triggering led to short-term increases in bladder and detrusor pressure (arrowheads in **A**, **B**, **C**, **D**) with minor subsequent urine losses at higher pressures (<35 cmH2O). The EMG signal was sound according to the stimulation parameters and demonstrated correct electrode placement **(D)**, which was confirmed visually.

The control urodynamic measurement showed weak bladder responses during active filling in all animals under light sedation. Spontaneous voiding was only recorded in one animal (Figure 2A). Bladder responses were absent in all animals during general anaesthesia (Figure 2B). Multiple manual external triggering provoked no urine loss and, at higher intravesicular pressures of more than 20 cmH2O, there was no micturition or passive urine loss. After onset of the pneumoperitoneum, there were no differences compared to the previous recordings due to a lack of any reaction of the bladder. Spontaneous micturition or passive urine loss could not be provoked by active filling or manual external triggering of the bladder (Figure 2C). Passive minor urine loss eventually occurred after stronger manual compression (<35 cmH2O) of the bladder, but it stopped immediately after the compression ended. Drainage had to be performed actively. During unilateral electrical stimulation, no differences were observed in bladder responses. Triggering did not provoke an active bladder response, and no urine loss, either passive or active, was recorded during stimulation (Figure 2D).

### Histology

**Figure 3:**
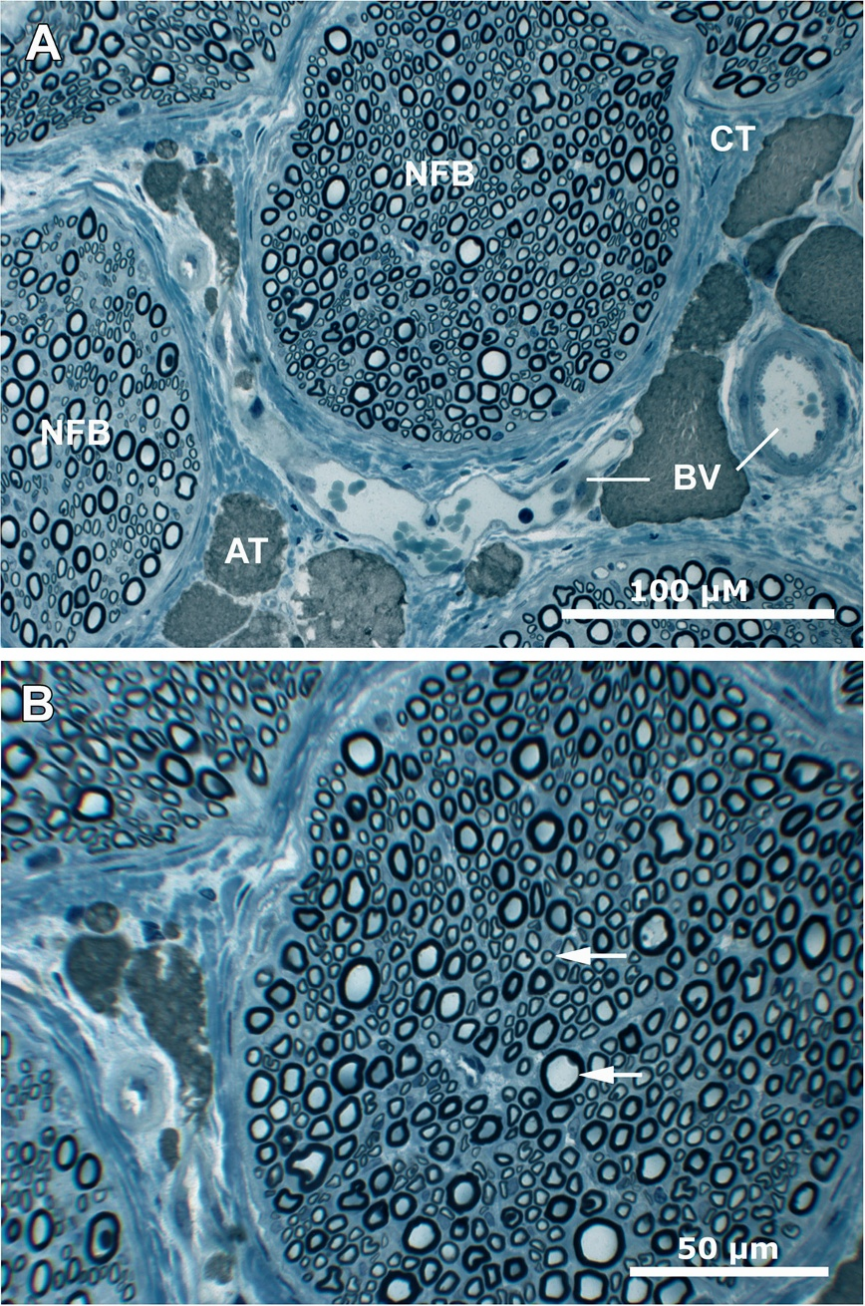
**Histology of the pudendal nerve.** Overview **(A)** of several nerve fibre bundles (NFB) embedded in dense perineurium comprising connective tissue (CT), adipose tissue (AT), and blood vessels (BV). The single nerve fibre bundles are densely packed with myelinated nerve fibres (arrows) and are surrounded by endoneurium **(B)**. Scale bars, 100 μm **(A)**, 50 μm **(B)**.

All pudendal nerves showed good preservation at the histological level (see Figure 3). The single dissected pudendal nerves varied in size from 0.2 to 1.1 mm at their largest expansion (see arrows in Figure 3). The nerve fibre bundles are embedded in preserved connective tissue, including adipose tissue and blood vessels. The single nerve fibre bundles were densely packed with myelinated nerve fibres and were surrounded by endoneurium that appeared to be homogenous.

### General discussion

Laparoscopy is a minimally invasive surgical technique that can be used for electrode implantation and for neuromodulation of the targeted visceral organs (Possover 2010; Possover et al. 2007, 2010; Rabischong et al. 2011). Currently, conventional neuromodulation is performed using a percutaneous approach in which a tined lead electrode is implanted near the target nerve using neuro-physiological monitoring (Spinelli et al. 2003). However, one disadvantage of a percutaneous approach is the absence of direct optical control for electrode placement; indeed, placement must be monitored using fluoroscopy and X-ray imaging.

The pig was chosen as the best animal model for this study because the anatomical, physiological, and pharmacological characteristics of porcine bladder function are considered most similar to those of humans (Jorgensen et al. 1983; Mills et al. 2000; Dalmose et al. 2005; Bossowska et al. 2009; Jensen et al. 2009). The basic requirements for laparoscopy in pigs are generally similar to those in humans, further supporting the appropriateness of the use of a pig model. Laparoscopy in pigs has been performed successfully in the past (Rabischong et al. 2011), and the sizes of the single selected peripheral nerves that innervate the bladder are comparable to those in humans and are therefore suitable for electrode implantation and chronic neuromodulation.

Due to advances in laparoscopic techniques and instrumentation, identification and exposure of the main somatic and autonomic nerves in the pelvis is now feasible using laparoscopy (Rabischong et al. 2011). Laparoscopic nerve location with subsequent electrode implantation has been successfully performed previously (Possover et al. 2007, 2010; Possover 2010; Rabischong et al. 2011), but this report is the first to describe standard laparoscopic access to the pudendal nerve in the pig model.

Laparoscopic placement may lower the risk of electrode migration due to more precise, diligent, and optically-controlled placement along the target nerve. Laparoscopic electrode implantation cannot be compared with the standard technique used for conventional sacral neuromodulation, as the latter involves accessing the S3 roots using a transforaminal approach. However, the location of the pudendal nerve makes a percutaneous procedure, either from the posterior or via the perineum, more difficult. The correct placement of the electrode using one’s knowledge of anatomical structures and “blindly” implanting the electrode at the nerve site with the help of neuro-physiological monitoring is not always feasible. Therefore, stimulation responses can vary greatly between patients. Accordingly, visually monitoring electrode placement using laparoscopy may be a preferable approach, even if it is slightly more invasive.

An empty bowel and bladder are important for this approach to the nerve, since faeces or a full bladder may hamper access to the pudendal nerve and make electrode implantation difficult and time consuming. Therefore, fasting for at least 24 h prior to surgery and continuous drainage of the bladder are crucial for the success of the surgery.

The tined lead electrodes that were used for this study and that are constructed for percutaneous implantation use only (Grill and Mortimer 2000) turned out to be mostly inappropriate for laparoscopic handling. However, this did not influence laparoscopic placement of the electrode. On the contrary, the tip of the electrode and particularly its shape and stiffness were helpful in guiding the electrode along the nerve and in pushing it forward with sound contact with the nerve. Sound contact was established with at least three to four poles, which is considered more than sufficient for excellent nerve stimulation. As described previously by other groups, neural cuff electrodes seem more appropriate for laparoscopic implantation (Grill and Mortimer 2000; Romero et al. 2001; Navarro et al. 2005; Vince et al. 2005a, [b]; Thil et al. 2007). To date, cuff electrodes are the most investigated type of implantable electrodes (Navarro et al. 2005; Rabischong et al. 2011). The potential risks of cuff electrodes include compression of the nerve, erosion, and continuous physical irritation of the nerve. The possible morphological changes in the nerve during implantation and chronic stimulation merit further investigation and, to date, only the obturator and sciatic nerves have been investigated (Vince et al. 2005a, [b]; Thil et al. 2007).

The absence of a bladder response before and during stimulation of the pudendal nerve during urodynamic measurements in the course of the surgery was not optimal. However, pudendal stimulation was observed as reflected by perianal skeletal muscle contractions during the two stimulation sets. In general, the absence of micturition even during light sedation once more underlines the fact that anaesthesia interferes with micturition (Matsuura and Downie 2000). Data from all animal models consistently show suppressed micturition during general anaesthesia, making intraoperative urodynamic measurements unnecessary. Moreover, the utility of urodynamic analyses of acute electrical stimulation of peripheral nerves is questionable, as anaesthesia suppresses or at least alters the bladder response. This calls into question the reliability of such data, and acute pudendal nerve stimulation itself may not accurately reflect the potential effectiveness of chronic stimulation. The continuous presence of a pneumoperitoneum during laparoscopic surgery amplifies these effects. Accordingly, chronic stimulation with regular follow-up analysis is needed to learn more about the potential interference of neuromodulation with bladder function.

Laparoscopic pudendal neuromodulation may facilitate the development of alternative neuromodulation methods that could be useful when sacral neuromodulation fails, when there are anatomical abnormalities, or for pain patients with potential involvement or pathology of the pudendal nerve. Furthermore, in terms of functional urology, specific bladder disorders cannot be treated exclusively by S3 modulation. Thus, access to additional peripheral nerves may be needed to ensure therapeutic success. The pudendal nerve is one of the most important peripheral nerves to be targeted for treating specific bladder disorders, and electrode implantation using laparoscopy could be the best surgical approach for accessing this nerve and for implanting an appropriate electrode for chronic neuromodulation.

## Conclusions

The aim of this study was to create a standard laparoscopic approach for localisation of the pudendal nerve and implantation of an electrode at the nerve site with subsequent stimulation and urodynamic and electromyographic evaluation of the urinary bladder and perineal skeletal muscles. This study showed that in an appropriate animal model, i.e. the pig, standardised laparoscopic localisation of the pudendal nerve and placement of an electrode at the nerve site under optical control is feasible, reproducible, and safe. Furthermore, this approach is suitable for electrode implantation and subsequent chronic pudendal neuromodulation using an appropriate type of electrode. Urodynamic measurements are challenging during anaesthesia as narcotic substances most likely influence bladder physiology. A laparoscopic approach may be an alternative to the conventional percutaneous approach for direct electrode implantation at any peripheral nerve target for neuromodulation purposes, such as for bladder dysfunction and chronic pelvic pain. With this aim, further animal studies have to be conducted to re-evaluate and further optimise the surgical technique and engineering, and electrode types must be tested. In addition, chronic functional stimulation outcomes must be assessed in studies to make this technique accessible to patients suffering from underlying clinical diseases.

## Methods

### Pilot study

In a pilot cadaveric study (data not shown), two female adult Large White pigs were sacrificed and exsanguinated using standard procedures in the presence of a veterinarian. The anatomical conditions were analysed and documented photographically in an open autopsy to plan a possible standard laparoscopic access route to the pudendal nerve.

### Animals and anaesthesia

In the main study, laparoscopy was performed on 10 female PIC-variety farm pigs (4 months old, body weight 22–30 kg). All procedures were performed in accordance with Romanian law pertaining to the care and use of laboratory animals and in accordance with the European Community Guidelines for the use of experimental animals. Ethical approval was obtained from the Ethics and Deontology Committee for Research on Animals at the University of Medicine and Pharmacy Timisoara. Before surgery, the animals were deprived of food for 24 hours but had access to water *ad libitum*. Animals were pre-anaesthetised intravenously with xylazine 2 mg/kg and ketamine 15 mg/kg. Anaesthesia was induced via intravenous administration of thiopentalum 7 mg/kg. After oral endotracheal intubation, 1% isoflurane carried by oxygen was used to maintain general anaesthesia throughout surgery. A manually recorded Anaesthesia and Intraoperative Animal Monitoring Record was used to document each animal’s vital signs, anaesthesia, and intravenous fluid administration during the surgery.

### Surgery

**Figure 4:**
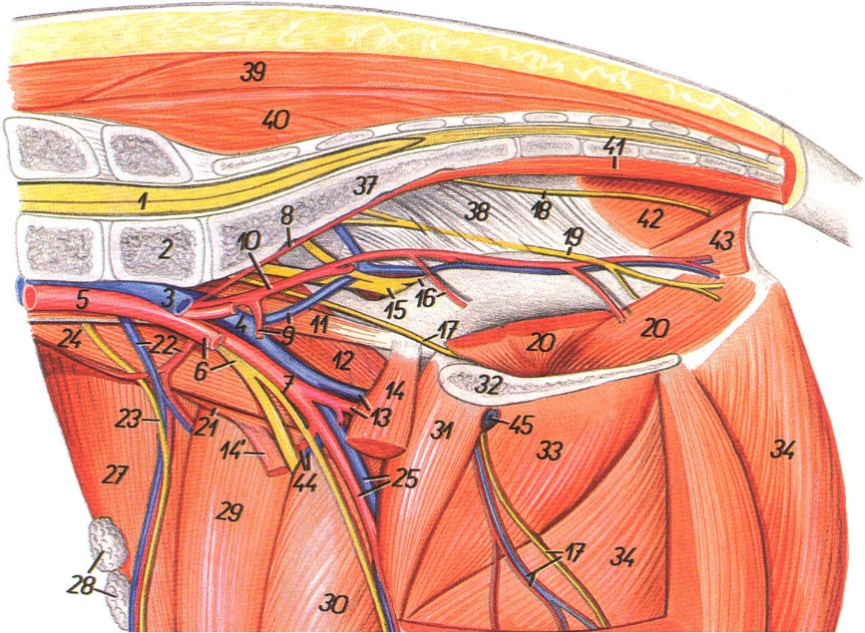
**Schematic drawing of the anatomy of the small pelvis of pigs.** Blood vessels and peripheral nerves that are relevant to the current study are highlighted. In particular, No. 9 and 10 corresponds to *A.* and *V. iliaca interna*, while no. 17 corresponds to the obturator nerve, no. 15 corresponds to sciatic nerve and no. 19 corresponds to the pudendal nerve (Popesko 1977). This illustration was published with permission from Enke Publishing, Stuttgart, Germany, and from Priroda Publishing, Bratislava, Slovakia.

The pneumoperitoneum was created using a Veress needle two fingers breadth below the umbilicus. Insufflation with CO_2_ was carried out until an intra-abdominal pressure of 12 mmHg was achieved. Two 12-mm suprapubic ancilliary trocars were placed (under direct vision) both caudal and lateral to the midline in a triangular position. The anatomical landmarks were defined in a 30° contralateral position. All surgical procedures were performed using standard laparoscopic instruments, including a 0° camera, graspers, and scissors (Karl Storz, Tuttlingen, Germany). The laparoscopy procedure started by localising the bifurcation of the internal iliac artery and vein, followed by dorsal dissection of the fascia to find the superficially located obturator nerve. Next, the pelvic sidewall was followed until the broad ligament of the pelvis was reached. The superficial layer of the ligament was incised and opened bluntly, and the pudendal nerve was located right underneath (Figure 4). The pudendal nerve was detached carefully from the surrounding tissue for a length of 20 to 30 mm. A tined lead electrode (percutaneous lead, Model 3887, Medtronic Inc.) was inserted through the ipsilateral trocar leaving the very end of the electrode on the outside for subsequent stimulation after reinsertion of the trocar. The electrode was placed in parallel to the pudendal nerve under optical guidance, and contact with the nerve was confirmed visually before starting stimulation.

### Catheterisation and urodynamics

Prior to laparoscopy, a 7-french air catheter was transurethrally inserted into the bladder (15 cm into the bladder) during light sedation. Two perineal surface electrodes were placed for electromyography recordings, while a null electrode was placed on a non-responsive skeletal muscle (*M. gracilis*). There were no active skeletal muscle contractions due to anaesthesia, so the abdominal pressure was not recorded. Correct placement of the catheter was verified by irrigation and urine backflow after insertion. Before starting the experiments, the bladder was drained manually using a syringe. Bladder pressure (cmH_2_O) was recorded during filling with 50 ml/min physiological warm (37°C) sodium chloride solution. The bladder was filled to its estimated maximum capacity of 200 ml. Electromyography recordings were conducted in parallel. The evaluations were performed twice, and the bladder was drained completely in between the two recordings. Two additional electromyography measurements were performed, once during general anaesthesia and one after the pneumoperitoneum was established. During surgery, the urinary bladder was allowed to drain continuously via a catheter. After placement of the electrode alongside the pudendal nerve and filling the bladder to 80% of its maximum capacity (160 ml), unilateral neurostimulation was performed starting with 210 μs, 10 Hz, and 10 V for 15 minutes, followed by a 5-minute break and subsequent stimulation with 450 μs, 50 Hz, and 10 V for another 15 minutes. The stimulation parameters were determined using a standard protocol for treating bladder retention or overactivity that is wide spread and routinely performed in patients treated with sacral neuromodulation therapy in our clinics. During these recordings, the bladder was regularly triggered to provoke bladder reactions. Triggering was performed until the onset of the surgery (after creation of the pneumoperitoneum) using manual external compression of the abdomen or after the onset of surgery using pressure applied by a grasper using optical guidance via a camera. The applied pressure was monitored by the urodynamic unit to ensure that it did not exceed 40 cmH_2_O. The measurement was repeated for the pudendal nerve on the opposite side after relocation of the electrode. After assessment of the electrode placement and after the final urodynamic recordings, the pudendal nerves were bilaterally dissected and fixed for histological analysis in 2.5% glutaraldehyde in 0.15 M sodium cacodylate buffer pH 7.4. The samples were post-fixed in 4% osmium tetroxide and, after a series of alcohol dehydration steps, embedded in epoxy resin for semi-thin sectioning. The sections were stained with methylene blue and examined under a light microscope.
